# Bioavailability as Proof to Authorize the Clinical Testing of Neurodegenerative Drugs—Protocols and Advice for the FDA to Meet the ALS Act Vision

**DOI:** 10.3390/ijms251810211

**Published:** 2024-09-23

**Authors:** Sarfaraz K. Niazi

**Affiliations:** College of Pharmacy, University of Illinois, Chicago, IL 60612, USA; sniazi3@uic.edu

**Keywords:** ALS Act, neurodegenerative disorders, novel clinical trials, transcytosis, rare diseases, alternative delivery systems

## Abstract

Although decades of intensive drug discovery efforts to treat neurodegenerative disorders (NDs) have failed, around half a million patients in more than 2000 studies continue being tested, costing over USD 100 billion, despite the conclusion that even those drugs which have been approved have no better effect than a placebo. The US Food and Drug Administration (FDA) has established multiple programs to innovate the treatment of rare diseases, particularly NDs, providing millions of USD in funding primarily by encouraging novel clinical trials to account for issues related to study sizes and adopting multi-arm studies to account for patient dropouts. Instead, the FDA should focus on the primary reason for failure: the poor bioavailability of drugs reaching the brain (generally 0.1% at most) due to the blood–brain barrier (BBB). There are several solutions to enhance entry into the brain, and the FDA must require proof of significant entry into the brain as the prerequisite to approving Investigational New Drug (IND) applications. The FDA should also rely on factors other than biomarkers to confirm efficacy, as these are rarely relevant to clinical use. This study summarizes how the drugs used to treat NDs can be made effective and how the FDA should change its guidelines for IND approval of these drugs.

## 1. Introduction

It is reported on the NLM’s ClinicalTrials.gov Beta (beta.clinicaltrials.gov; accessed 25 August 2024) that 109 clinical trials on Alzheimer’s disease (AD) were discontinued during the last decade [1]. These terminations were attributed to several factors, including insufficient funding, interrupted visits caused by COVID-19, challenges in enrolling participants, safety concerns, delayed recruitment of eligible patients, inadequate study design to meet the trial’s objectives, conflicting safety or efficacy data from other studies, unfavorable risk–benefit ratio, and inappropriate dosage settings [1].

A comprehensive analysis of 33,689 individuals from 42 studies found no significant difference between anti-Aβ medications and the placebo in the Alzheimer’s Disease Assessment Scale–Cognitive Subscale (ADAS-Cog). However, anti-Aβ medications were linked to a significant risk of adverse events [ADAS-Cog: Mean Differences = −0.08 (−0.32 to 0.15), *p* = 0.4785; and Adverse Events: Relative Risk = 1.07 (1.02 to 1.11), *p* = 0.0014] [2].

An alternative study presented findings from 33 randomized controlled trials, including 21,087 patients and eight monoclonal antibodies (mAbs). Despite the substantial increase in the risks of adverse events and reactions associated with immunotherapies, the data indicate that monoclonal antibodies (mAbs) can successfully enhance the cognitive function of patients with mild and moderate Alzheimer’s disease (AD) [2]. Based on the NMA, aducanumab showed the highest probability for substantially strengthening cognitive and clinical evaluations, including statistically improved MMSE and CDR-SB. Donanemab showed statistically improved ADAS-Cog and PET-SUVr, while lecanemab showed improved ADCS-ADL measures [3].

These research findings indicate that anti-Aβ medications have little impact on cognitive function in individuals with Alzheimer’s disease. Yet, monoclonal antibodies directed against Aβ can postpone cognitive deterioration in Alzheimer’s disease [2].

Monoclonal antibodies outperformed the placebo in delaying cognitive deterioration as measured by the ADAS-Cog, Clinical Dementia Rating–Sum of Boxes (CDR-SB), Mini-Mental State Examination (MMSE), and Alzheimer’s Disease Cooperative Study–Activities of Daily Living (ADCS-ADL), without increasing the risk of adverse events [ADAS-Cog: MDs  =  −0.55 (−0.89 to 0.21), *p* = 0.001; CDR-SB: MDs  =  −0.19 (−0.29 to −0.10), *p* <  0.0001; MMSE: MDs  =  0.19 (0.00 to 0.39), *p* =  0.05; and ADCS-ADL: MDs  =  1.26 (0.84 to 1.68), *p* <  0.00001]. Intravenous immunoglobulin and γ-secretase modulators (GSMs) exacerbated cognitive decline in patients with CDR-SB [mean difference [MD = 0.45 (0.17 to 0.74), *p* = 0.002]. Nevertheless, their safety profiles in patients with AD have been deemed satisfactory. Gamma-secretase inhibitors (GSIs) exacerbated cognitive deterioration in ADAS-Cog, MMSE, and ADCS-ADL assessments. The administration of BACE-1 inhibitors exacerbated cognitive decline as measured by the Neuropsychiatric Inventory (NPI). GSI and BACE-1 inhibitors raised safety-related issues. Existing research has not indicated cognitive function as being affected by active Aβ immunotherapy, MPAC, or tramiprosate; however, tramiprosate has been linked to severe side effects [4].

The FDA has approved only three products: aducanumab (withdrawn), donanemab, and lecanemab (approved in the US and rejected by the EMA for safety concerns) [5]. The efficacy of other antibodies, such as solanezumab, crenezumab, and gantenerumab, remains to be demonstrated. Despite showing reductions in Aβ plaques, *bapineuzumab* studies were terminated as it lacked treatment effects in two phase 3 trials. Side effects related to vascular Aβ were also raised as a concern [6]. In July 2024, Pfizer withdrew its gene therapy fordadistrogene movaparvovec due to a lack of efficacy [7]. As no therapeutic strategy has been proven successful in curing any NDs, many scientists question whether protein aggregation is central to the etiology of NDs or whether it is, instead, a manifestation of other underlying causes [8,9].

Statistical significance has not been achieved in many clinical trials, such as the case of Qalsody (used for treating ALS associated with mutations in the SOD1 gene), for which a study involving 108 participants showed no significance in slowing disease progression as measured by the ALS Functional Rating Scale-Revised (ALSFRS-R) over 28 weeks. Yet, it was approved due to significant reductions in biomarkers associated with neuronal injury [10].

While biomarkers in neurodegenerative disease treatment have become pivotal in assessing therapeutic interventions, their reliability in predicting clinical outcomes has often needed to be more consistent. Common biomarkers include cerebrospinal fluid (CSF) tau and amyloid-beta levels, which are primarily used in Alzheimer’s disease (AD), and the neurofilament light chain (NfL), a marker for neuronal damage across various neurodegenerative conditions. For instance, in the EMERGE and ENGAGE trials of the anti-amyloid drug aducanumab, significant reductions in amyloid-beta were observed, and these changes were associated with corresponding biomarker alterations; however, this did not consistently translate to cognitive improvements, resulting in significant debate regarding the drug’s clinical efficacy. Similarly, the EXPEDITION trials involving solanezumab, another anti-amyloid therapy, demonstrated modest reductions in amyloid plaques but failed to show any significant clinical benefits, questioning the utility of amyloid-beta as a reliable biomarker for therapeutic success [11]. In the case of NfL, while it has been effective in tracking disease progression in disorders such as multiple sclerosis and amyotrophic lateral sclerosis, studies such as those involving the anti-SOD1 therapy in ALS failed to show a clear correlation between NfL reduction and clinical improvement, raising concerns about its reliability as a surrogate endpoint [12]. These examples underscore the complexity of neurodegenerative diseases and highlight the need to integrate multiple biomarkers and clinical endpoints in order to assess treatment efficacy accurately.

However, despite existing evidence, the search for ND treatment entities remains active, with over half a million studies currently underway to establish efficacy (phase I–IV), as detailed in Table 1.

Since 1995, cumulative private expenditures on clinical stage AD R&D have been estimated at USD 42.5 billion, with the most significant costs (57%; USD 24,065 million) incurred during phase 3. Approximately 184,000 participants were registered or are currently enrolled in clinical trials [13]. At present, there are 2270 studies enrolling 786,241 patients in the various development phases listed, which should cost over USD 100 billion, almost all of which will likely go to waste. Appendix A lists prominent drugs of all classes that have failed in clinical trials for the treatment of NDs.

## 2. Bioavailability

Monoclonal antibodies have recently been developed to treat neurodegenerative diseases (NDs), which operate by crossing the blood–brain barrier (BBB). Nevertheless, the brain’s bioavailability of these substances is commonly reported to be approximately 0.1% of the quantities reached in the bloodstream. Numerous studies have reported that central nervous system (CNS) exposure to circulating biologics is restricted to 0.1 to 0.4% of the corresponding quantities in the bloodstream. However, some estimates for transporting immunoglobulins into neural tissue are much lower [14].

Therefore, maximizing the concentration of big molecules administered peripherally in the brain is necessary to attain the desired level of involvement required for a therapeutic response. Nevertheless, this method is precarious, with the potential for heightened adverse reactions. One primary adverse impact of anti-Aβ antibodies is the occurrence of “amyloid-related imaging abnormalities” (ARIAs), which are detected by MRI and can be categorized into two subtypes: edema (ARIA-E) and hemorrhage (ARIA-H) [14].

### 2.1. Barriers to Bioavailability

**Figure 1:**
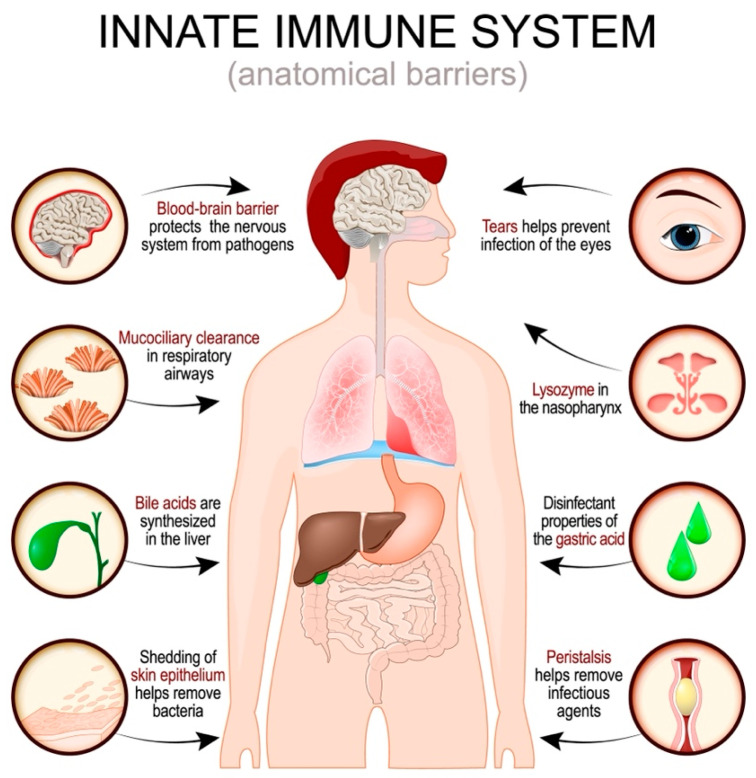
Human innate immune system [Shutterstock-licensed image, Shutterstock_2146447853].

Some anatomical barriers in the body serve as crucial immuno-protective mechanisms, acting as the first line of defense against pathogens and harmful substances (Figure 1).

One of the primary anatomical barriers is the skin, which is the body’s largest organ and plays a critical role in protecting against environmental threats. The skin comprises multiple layers, including the epidermis, which is fortified with a keratinized layer which provides a formidable, impermeable barrier against pathogens. The skin’s surface also hosts a diverse microbiome and produces antimicrobial peptides that inhibit microbial growth. Its slightly acidic pH and secretion of sebum, which contains antimicrobial lipids, further enhance its defensive capabilities. Immune cells located in the epidermis, such as Langerhans cells, contribute to the immune surveillance and early detection of pathogens [15].

Mucous membranes, lining the respiratory, gastrointestinal, and urogenital tracts, also serve as vital anatomical barriers [16]. These membranes are covered with mucus, a viscous fluid which traps microbes and other particles, preventing their entry into the body. The mucus contains immunoglobulins, particularly IgA, which are crucial in neutralizing pathogens. Cilia, hair-like structures present on the surface of some mucous membranes, work in concert with the mucus by moving trapped particles towards exits such as the mouth or nose, facilitating their removal from the body. This mucociliary clearance mechanism is especially important in the respiratory tract, where it helps prevent infections by expelling inhaled pathogens.

In addition to the skin and mucous membranes, the blood–brain barrier (BBB) represents a specialized anatomical barrier that protects the central nervous system [17]. The BBB comprises tightly joined endothelial cells, astrocyte end-feet, and a basement membrane, which restrict the passage of pathogens, toxins, and even specific immune cells into the brain. This selective permeability ensures that the neural environment remains stable and free from potential infections or inflammatory responses that could disrupt brain function. Similarly, the gastrointestinal barrier, comprising the intestinal epithelium and its associated mucus layer, acts as a critical means of defense in the gut [18]. It prevents the entry of pathogens while permitting the absorption of nutrients. This barrier is maintained by tight junctions between epithelial cells, antimicrobial peptides, and a complex immune system that includes gut-associated lymphoid tissues [19].

Other anatomical barriers include the ocular surface, which is protected by tears containing lysozyme and other antimicrobial factors, and the genitourinary tract, which utilizes acidic pH, mucus, and regular flushing through urination to prevent microbial colonization [20]. Collectively, these anatomical barriers provide essential, multilayered protection, working together to stop the invasion of pathogens and maintain the body’s overall health and immune homeostasis.

Multiple studies have confirmed that, with the current strategies, Huntington’s disease targets in the brain cannot be reached [21], with similar findings for Parkinson’s disease [22], spinocerebellar ataxia (SCA) [21], frontotemporal dementia (FTD) [23], ALS, multiple sclerosis (MS) [24], Alzheimer’s disease [25], and Creutzfeldt–Jakob disease (CJD) [26].

In addition to the blood–brain barrier (BBB) and the cerebrospinal fluid (CSF) barrier, the central nervous system (CNS) is also protected and regulated by several other obstacles. These barriers are critical in maintaining the CNS environment, ensuring proper neuronal function and protecting the brain and spinal cord from harmful substances. They collectively contribute to the homeostasis and protection of the CNS, each serving specific roles in maintaining the integrity and functionality of the nervous system. These barriers each have a different anatomy, and forcing a drug to cross these barriers requires a focused approach to increase its bioavailability in the targeted tissue. Understanding these barriers is crucial for developing strategies for drug delivery to the CNS and addressing various neurological disorders.

### 2.2. Blood–Brain Barrier (BBB)

**Figure 2:**
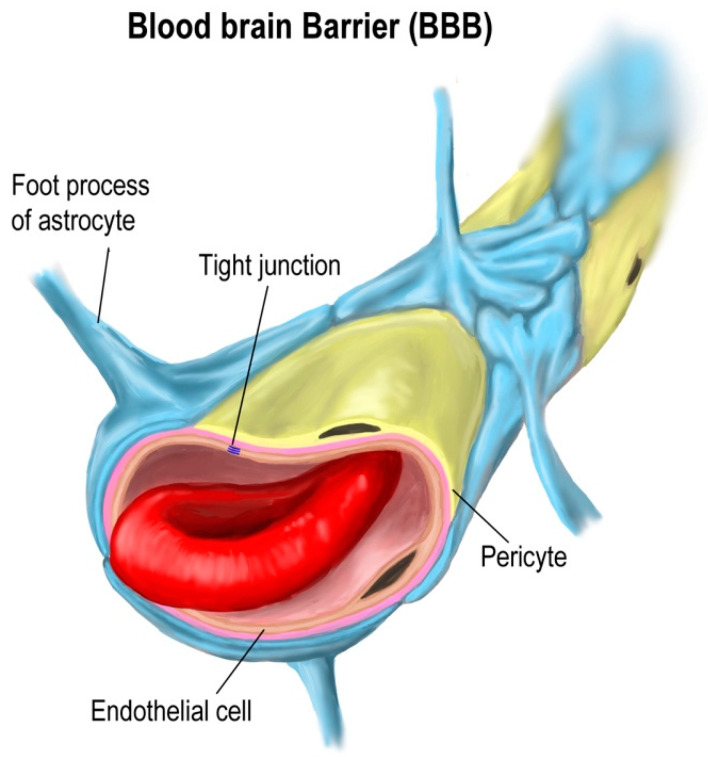
The blood–brain barrier is the strongest barrier, protecting the brain through multiple mechanisms [Shutterstock-licensed image, shutterstock_1933594793].

The BBB is located at the level of the brain capillaries and is formed by endothelial cells that are tightly joined together by tight junctions, creating a barrier with highly selective permeability (Figure 2).

The BBB’s primary function is to control the entry of substances from the bloodstream into the brain, allowing essential nutrients to pass through while blocking potentially harmful compounds. Additionally, the BBB maintains the brain’s microenvironment, which is crucial for proper neuronal function. The BBB utilizes various mechanisms to transport substances, including passive diffusion for small lipophilic molecules, active transport for essential nutrients, and receptor-mediated transcytosis for specific proteins such as transferrin [17,27].

### 2.3. Blood–Cerebrospinal Fluid Barrier (BCSFB)

In contrast to the BBB, the blood–CSF barrier is primarily located at the choroid plexus, a network of capillaries surrounded by epithelial cells within the brain’s ventricles. These cells have tight junctions like those of the BBB, but specialize in different functions. The BCSFB regulates the exchange of substances between the blood and the CSF, controls the composition of the CSF, and facilitates the removal of waste products from the brain [28].

The CSF is produced by these epithelial cells and flows through the ventricles and around the brain and spinal cord. The CSF barrier’s primary function is to regulate the composition of the CSF, remove waste products from the brain, and provide a cushioning effect to protect the brain and spinal cord. It also helps maintain intracranial pressure [28]. The epithelial cells of the choroid plexus exhibit different permeability properties compared to those in the BBB. These cells actively transport certain ions and nutrients into the CSF while restricting the entry of larger molecules [29].

Several differences exist between the BBB and the CSF barrier. First, the BBB is primarily formed by endothelial cells, while the CSF barrier is formed by the choroid plexus’ epithelial cells. Second, the BBB is more restrictive in terms of permeability than the CSF barrier, which allows the passage of a different set of molecules, reflecting its role in maintaining CSF composition. Third, while both barriers protect the brain, the BBB mainly regulates the brain’s microenvironment, and the CSF barrier primarily manages the composition of the CSF and waste removal [30].

These differences have significant implications for drug delivery. Targeting the BBB involves strategies including receptor-mediated transcytosis (e.g., using transferrin), nanoparticle delivery systems, and chemical modification of drugs to enhance their lipophilicity [31]. In contrast, drug delivery to the CSF often involves direct administration methods, such as intrathecal injection, due to the differences in permeability and transport mechanisms compared to the BBB [32]. Understanding these differences is crucial for developing effective CNS drug delivery strategies. Diseases associated with the BCSFB include meningitis, Chiari malformation, and subarachnoid hemorrhage.

While transferrin-mediated delivery can enhance drug transport to the brain, it may be less effective for direct delivery into the CSF. Intrathecal administration, which involves direct injection into the CSF, bypasses the BBB and allows for higher drug concentrations in the CSF. This method is commonly used for treating conditions such as meningitis and certain cancers [32]. Therefore, different strategies might be necessary, such as intrathecal delivery or utilizing other target ligands specific to the choroid plexus.

The primary pathway for CSF absorption into the venous system is through the arachnoid villi and granulations in the superior sagittal sinus and other venous sinuses. These structures act as one-way valves, allowing CSF (and any dissolved substances, including drugs) to enter the venous blood while preventing backflow. Evidence shows that CSF can also be absorbed into the lymphatic system, particularly through the nasal lymphatics associated with the cribriform plate. This can lead to drug molecules entering the lymphatic circulation and, eventually, the systemic bloodstream. Drugs can also diffuse through the ependymal lining of the ventricles and the pial surfaces into the brain parenchyma, then into the surrounding blood vessels, as they can move from the CSF into the perivascular spaces around blood vessels and then diffuse into the systemic circulation.

### 2.4. The Blood–Retinal Barrier (BRB)

This barrier is in the eye and consists of two components: the inner BRB, formed by retinal endothelial cells with tight junctions, and the outer BRB, formed by retinal pigment epithelial cells. Like the BBB in the brain, it protects the retina by regulating the exchange of substances between the blood and the retinal tissue. It maintains the retinal environment, which is essential for proper visual function [33]. The blood–retinal barrier shares similarities with the BBB regarding tight junctions and selective permeability. Transferrin receptors are also present in the retina, making transferrin a potential carrier for drug delivery to the eye. However, specialized carriers such as peptides or antibodies that target specific retinal receptors might offer better efficacy and selectivity [33]. Diseases associated with the BRB include diabetic retinopathy, where hyperglycemia leads to BRB breakdown, causing the following: retinal swelling and vision loss; age-related macular degeneration (AMD), where BRB dysfunction contributes to the accumulation of drusen and retinal degeneration; retinal vein occlusion, where the blockage of retinal veins can increase BRB permeability, leading to macular edema; and uveitis, in which the inflammation of the uveal tract can compromise the BRB, causing fluid leakage and damage to retinal tissues [33].

### 2.5. Arachnoid Barrier

The arachnoid mater forms the arachnoid barrier, one of the three meninges covering the brain and spinal cord. The arachnoid cells are tightly packed, creating a barrier between the subarachnoid space (where CSF flows) and the dura mater. This barrier prevents the leakage of CSF into the surrounding tissues and helps maintain the pressure and composition of the CSF [34]. The arachnoid barrier is less permeable and primarily prevents CSF leakage into the surrounding tissues. Overcoming this barrier typically involves invasive methods such as direct CSF administration, rather than receptor-mediated delivery. Thus, transferrin might not effectively target this barrier [34]. Diseases associated with the arachnoid barrier include the following: arachnoid cysts, fluid-filled sacs between the arachnoid and brain or spinal cord which can disrupt normal CSF flow and barrier function; subdural hematoma, where bleeding under the dura mater can press on the arachnoid barrier, leading to neurological symptoms; and meningiomas, tumors originating in the meninges, which can affect the integrity of the arachnoid barrier and the surrounding tissues.

### 2.6. Ependymal Barrier

This barrier is formed by ependymal cells lining the brain’s ventricles and the spinal cord’s central canal. These cells have cilia and microvilli that aid in the circulation of CSF. As for its function, while the ependymal layer is not as tightly sealed as the BBB or BCSFB, it plays a role in the exchange of substances between the CSF and the brain parenchyma, and helps circulate the CSF throughout the ventricular system [35]. The ependymal barrier is more permissive compared to the BBB and the CSF barrier. However, as it lines the ventricles and central canal, drugs need to be delivered via the CSF. Intraventricular or intrathecal administration is more appropriate than transferrin-mediated delivery for targeting the ependymal barrier [35]. Diseases associated with the ependymal barrier include the following: ependymitis, involving the inflammation of the ependymal cells—often due to infection—which can impair CSF flow and barrier function; hydrocephalus, denoting abnormal CSF accumulation; and ependymoma, tumors arising from ependymal cells which can disrupt normal barrier function and CSF flow [35].

### 2.7. Glia Limitans

A thin layer of astrocytic end-feet lines the surface of the brain and spinal cord beneath the pia mater. This barrier protects the CNS by regulating the movement of molecules between the subarachnoid space and the brain parenchyma. It also participates in the immune response within the CNS [36]. The glia limitans, formed by astrocytic end-feet, regulates the exchange of molecules between the brain parenchyma and the subarachnoid space. This barrier does not have transferrin receptors, so transferrin is not a suitable carrier. Approaches such as local delivery systems or cell-penetrating peptides might be more effective [37]. Relevant diseases include the following: astrogliosis, involving reactive changes in astrocytes which can disrupt the glia limitans, affecting the barrier between the brain parenchyma and the subarachnoid space; cerebral edema, swelling in the brain which can compress the glia limitans, leading to barrier dysfunction; and neuroinflammation, which can damage the glia limitans, thus impacting its protective function.

### 2.8. Blood–Nerve Barrier (BNB)

This barrier is found in peripheral nerves and is formed by tight junctions between endothelial cells of the endoneurial microvessels. It regulates the microenvironment of peripheral nerves by controlling the exchange of substances between the blood and the endoneurial fluid, protecting nerves from potentially harmful substances [38]. While transferrin receptors are present, other carriers or delivery methods, such as lipid-based nanoparticles or direct injection into peripheral nerves, offer better results. Relevant diseases include the following: diabetic neuropathy, where high blood sugar levels can damage the BNB, leading to nerve damage and pain; Guillain–Barré Syndrome, in which the immune-mediated attack on peripheral nerves can involve BNB disruption; chronic inflammatory demyelinating polyneuropathy (CIDP), where the inflammatory damage to the BNB contributes to chronic nerve dysfunction; and peripheral neuropathies, which can cause BNB breakdown, leading to nerve damage and sensory deficits.

## 3. Forward Path

The Fc region of antibodies plays a significant role in treating neurodegenerative disorders due to its involvement in immune modulation and pathogenic protein clearance. This region interacts with Fc receptors (FcRs) on immune cells, such as microglia and macrophages, enhancing phagocytosis and removing neurotoxic aggregates like amyloid-beta and tau, which are implicated in Alzheimer’s disease and other neurodegenerative conditions [39]. Furthermore, the Fc region can activate the complement system, further facilitating the clearance of damaged cells and protein aggregates [40]. These immune functions, including antibody-dependent cellular cytotoxicity (ADCC) and antibody-dependent cellular phagocytosis (ADCP), are essential in targeting and clearing pathological proteins that accumulate in neurodegenerative disease patients [41].

However, the relevance of the Fc region can vary depending on the context. For instance, treatments comprising antibody fragments such as Fab, scFv, or nanobodies do not contain Fc regions and are specifically used to target and neutralize pathological proteins without triggering immune effector functions. These fragment-based antibodies can achieve therapeutic effects without the risks associated with Fc-mediated immune activation, making them particularly suitable for conditions like Alzheimer’s disease where the direct inhibition or clearance of amyloid-beta is desired without additional immune responses [42]. In some neurodegenerative disorders, excessive immune activation can be detrimental. In diseases like multiple sclerosis or ALS, Fc-mediated functions might induce harmful inflammation, suggesting that antibodies without Fc regions could offer a safer therapeutic alternative [43]. Additionally, the size of the Fc region can hinder the transport of antibodies across the blood–brain barrier (BBB), reducing treatment efficacy in the central nervous system. Antibodies or fragments lacking Fc regions can potentially overcome this barrier more effectively, enhancing therapeutic potential [42]. Furthermore, in cases where intracellular or non-immune accessible targets are involved, such as alpha-synuclein in Parkinson’s disease, Fc-mediated immune functions may be less effective. Antibody fragments that can enter cells without immune activation are more advantageous in these contexts [43]. Additionally, Fc regions can inadvertently trigger adverse immune responses, such as antibody-dependent enhancement (ADE), which can worsen clinical outcomes, especially in autoimmune-related neurodegenerative diseases [39]. These insights underscore that, while the Fc region is beneficial in many scenarios, its utility can be context-dependent, and in certain cases, minimizing or eliminating the Fc region may lead to improved therapeutic outcomes, particularly when targeting intracellular proteins, enhancing BBB penetration, or reducing immune-related side effects.

In those instances where the Fc region can be removed, the optimization of molecular size is generally expected to improve bioavailability. Examples include single-chain variable fragment antibodies (scFvs), such as scFV-CH3 (minibodies), diabodies, single-domain antibodies (SdAbs), F(ab)2 fragments, F(ab) fragments, reduced IgG (rIgG), bispecific antibodies (BsAbs), and multi-specific antibodies, which all are a better option to improve bioavailability.

However, it is worth noting that, if an antibody is too small, it will be cleared rapidly through the kidneys, making it less effective. Antibodies that are too small offer better results when combined with receptor-mediated transcytosis (RMT), adsorptive-mediated transcytosis, and cell-mediated transcytosis [44,45].

Recombinant antibodies can be designed to specifically attach to the TfR, either directly or using transferrin. Optimal antibody engineering should aim for binding to a distinct epitope from Tf on the TfR to prevent disruption of the natural iron delivery process to the brain. Several TfR antibodies specifically attach to the apical domain of TfR enzymes [46,47]. The specific binding affinity for TfR should be moderate, within the nanomolar range [48]. An increased affinity may cause a reduced capacity of the antibody to separate from the TfR, resulting in its segregation into lysosomes for degradation [38]. In this context, the significance of the dissociation constant seems to surpass that of the association constant [48]. Insufficient affinity may result in an inadequate capacity of the antibody to attach to the TfR at the blood–brain barrier and, as a result, reduce effective transport to the brain [39]. The antibody should have a pH-dependent affinity, meaning that it binds effectively to the TfR at a physiological pH but dissociates at a lower pH in the early endosome [49].

In vivo binding was initially attempted using a monovalent anti-TfR compound based on Roche’s “brain shuttle” technology applied to gantenerumab (Trontinemab) (RG6102), which demonstrated an 8-fold improved CSF-to-serum ratio when compared to regular gantenerumab. The Fab fragment interacts explicitly with the transferrin receptor and bonds to the effector (Fc) domain of the gantenerumab monoclonal antibody. Thus, it undergoes endocytosis and then releases antibodies into the brain parenchyma [50]. Nevertheless, this method has many inherent disadvantages. First and foremost, the interaction with transferrin in living organisms will always be uncertain, contingent, and considerably risky.

Furthermore, the binding characteristic is determined by the arrangement of Fab, which relies only on naturally occurring linkers in the body. The non-cleaving linkers induce exocytosis when the conjugate formed inside the cell attaches to the receptor and re-enters the general circulation. This characteristic diminishes the therapeutic efficacy. Although it is expected that the binding of Fab to transferrin will enable iron binding, no guarantee exists that this will not impact the iron transport cycle. As previously mentioned, the linker between the antibody and its fragment must be capable of being cleaved. Therefore, after entry into the brain, the linker is severed, thus preventing exocytosis of either the antibody or the fragment.

However, if the RMT can be so effective, why is it not a primary approach to treating NDs? A reason can be the perceived understanding that binding antibodies with any other protein, such as transferrin, will inevitably interfere in their binding with the target protein. These misconceptions have been removed through simulation studies, showing that conjugating with the transferrin protein can even improve their binding to target proteins [51]; further, their binding with a portion of transferrin, N-lobe, and the scFvs of these antibodies have been studied such that developers can readily demonstrate their effects in animal studies, as proposed above.

### 3.1. Nanoparticles

Nanoparticles have emerged as a promising strategy for facilitating the delivery of therapeutic agents, particularly antibodies, across the blood–brain barrier (BBB) for the treatment of various neurodegenerative disorders. The BBB poses a significant challenge in neuropharmacology due to its selective permeability, which restricts the entry of most drugs into the brain [52]. Utilizing nanoparticles as carriers can enhance the transport and targeted delivery of antibodies by exploiting receptor- and adsorptive-mediated transcytosis [53].

For example, researchers have developed transferrin-modified nanoparticles that target the transferrin receptors abundantly expressed on the BBB. These nanoparticles have been used to deliver anti-beta-amyloid antibodies in Alzheimer’s disease models, significantly reducing the amyloid plaque burden and improving cognitive function [54]. Another approach involves using polymeric nanoparticles such as PLGA [poly(lactic-co-glycolic acid)] conjugated with glutathione, which resulted in increased brain penetration and the delivery of neuroprotective antibodies in Parkinson’s disease models, leading to a reduction in dopaminergic neuron loss and motor function improvement [55].

Liposomal nanoparticles modified with apolipoprotein E (ApoE) have been successfully employed to deliver antibodies targeting tau proteins, a hallmark of the pathology of Alzheimer’s. This system has been demonstrated to promote improved antibody accumulation in the brain and the subsequent inhibition of tau aggregation, resulting in neuroprotection and enhanced cognitive performance in animal models [56].

Recent studies have also explored the use of exosome-mimicking nanoparticles, which leverage the natural ability of exosomes to cross the BBB. These nanoparticles, loaded with therapeutic antibodies against alpha-synuclein, have shown potential in treating Parkinson’s disease through reducing the accumulation of toxic alpha-synuclein aggregates in the brain [57].

Moreover, the surface modification of nanoparticles with specific ligands, such as the angiopep-2 peptide, has been shown to further enhance BBB penetration and target specificity [55]. For instance, angiopep-2-modified gold nanoparticles have been used to deliver monoclonal antibodies against amyloid-beta, significantly reducing the amyloid levels and improving cognitive function in models of Alzheimer’s disease [54].

Nanoparticles can be engineered to cross the BBB and BSCB, providing a versatile platform for drug delivery. Liposomes and solid lipid nanoparticles can encapsulate drugs and target them in the CNS. They can be functionalized with ligands or antibodies for enhanced targeting and uptake [58]. Polymeric nanoparticles can be designed to release drugs in a controlled manner and can be modified to enhance BBB and BSCB penetration; examples include PLGA [poly(lactic-co-glycolic acid)] nanoparticles [59].

These advancements in nanoparticle technology underscore their potential in overcoming the challenges associated with BBB permeability, offering new avenues for the effective treatment of neurodegenerative diseases through the enhanced delivery of therapeutic antibodies.

### 3.2. Physical Means

Physical methods to enhance drug penetration across the blood–brain barrier (BBB) have gained significant attention, as they provide a direct and often more controllable means to facilitate drug delivery into the brain.

One of the most widely researched methods is focused ultrasound (FUS), which, when combined with microbubbles, can temporarily disrupt the BBB in a targeted and reversible manner. This technique works by inducing mechanical stress on the endothelial cells of the BBB, leading to the formation of transient openings through which therapeutic agents, including large molecules such as antibodies, can pass into the brain parenchyma [60]. FUS has been successfully applied in pre-clinical models to deliver drugs for the treatment of Alzheimer’s disease and glioblastoma, showing improved drug distribution and therapeutic efficacy [61]. FUS, in combination with microbubbles, can also temporarily disrupt the BSCB, allowing for the enhanced delivery of therapeutic agents directly to the CNS.

Another physical method involves the use of electromagnetic fields, which is called transcranial magnetic stimulation (TMS). TMS can modulate neuronal activity and has been shown to transiently increase BBB permeability, thereby enhancing the delivery of neurotherapeutic agents [62]. This method is non-invasive and has the advantage of being able to target specific brain regions with precision, which is particularly useful for the treatment of localized brain disorders [63].

In addition, intranasal delivery is an emerging technique that exploits the olfactory and trigeminal nerve pathways to bypass the BBB entirely. Through delivering drugs directly to the nasal cavity, this method allows for rapid transport to the brain, making it particularly suitable for delivering peptides, proteins, and nucleic acids [64]. Studies have demonstrated that the intranasal administration of therapeutic antibodies leads to significant brain accumulation and therapeutic effects in neurodegenerative disease models [65].

Moreover, convection-enhanced delivery (CED) is a method that involves the direct infusion of drugs into the brain’s interstitial space using a catheter. This technique creates a pressure gradient that drives therapeutic agents into the brain tissue, thus overcoming the limitations imposed by the BBB. CED has been explored for the delivery of chemotherapeutics, neurotrophic factors, and viral vectors for gene therapy, with promising results in clinical trials for conditions such as Parkinson’s disease and gliomas [66].

Drug delivery into the CSF via intrathecal injection bypasses the BBB and BSCB, providing a direct route to the spinal cord and brain [67]. This method is already used for ALS treatments, such as administering antisense oligonucleotides. These strategies and carriers are being actively researched and have significant potential to improve drug delivery in ALS, potentially enhancing the efficacy of therapeutic interventions for this challenging neurodegenerative disorder [68].

## 4. FDA Efforts

The FDA has been proactive in resolving scientific issues; in one case, the FDA conducted clinical trials to determine how its guidelines should be revised. Recent examples include FDA-conducted studies proving that all biosimilars could be interchangeable and introduced a new guideline in line with this change [69]. Other FDA efforts include introducing OMICS technology for the evaluation of new biological drugs [70]. These efforts continue the Orphan Products Grants Program (ORGP) introduced in 1983, facilitating the approval of more than 80 products [71]. The CDER’s Accelerating Rare Disease Cures (ARC) Program brings together CDER’s collective expertise and activities to provide a strategic overview and coordination of their rare disease activities [72]—mainly related to neurological disorders [73]—in compliance with the “Accelerating Access to Critical Therapies for ALS Act” [74], which required the FDA and the National Institutes of Health (NIH) to implement a public–private partnership to advance the understanding and foster the development of treatments for ALS and other rare NDs.

This act required the FDA to publish and implement a five-year action plan that allows it to award grants or contracts of up to 400 million USD over five years to cover the costs of research on and development of interventions intended to prevent, diagnose, mitigate, treat, or cure these diseases. 

On 24 June 2022, the FDA shared its “Action Plan for Rare Neurodegenerative Diseases including Amyotrophic Lateral Sclerosis: A five-year action plan” to meet its requirements under Section 4 of the Accelerating Access to Critical Therapies for ALS Act [75].

On 14 September 2022, the FDA and the NIH announced the launch of the Critical Path for Rare Neurodegenerative Diseases (CP-RND), a public–private partnership recognizing that, despite significant and sustained investments in drug development in neurological diseases, bringing transformative therapies to patients in need remains challenging for reason such as (but not limited to) an incomplete understanding of underlying disease progression and relevant sources of variability, inefficiencies and limitations in traditional clinical trial designs and analysis methods, and inadequate trial sizes and durations. These issues increase sponsors’ risk of engaging in late-stage drug development and impede the development of novel therapies. This collaboration of the FDA with C-Path has led to the development of tools to advance clinical trials for neurologic diseases and model-informed drug development (MIDD) tools for the design of clinical trials.

On 29 September 2023, the FDA announced that it had awarded over 16.9 million USD under the ALS Act. The FDA’s Center for Drug Evaluation and Research (CDER) collaborated with the non-profit association Critical Path Institute (C-Path) [76] to expedite the development of treatment modalities for Alzheimer’s Disease, Parkinson’s Disease, and Duchenne muscular dystrophy.

On 27 July 2024, the FDA announced the establishment of the “Rare Disease Innovation Hub” (the Hub), which has three primary functions [77]:

- The Hub will serve as a single point of connection and engagement with the rare disease community, including patient and caregiver groups, trade organizations, and scientific/academic organizations, for matters that intersect CDER and CBER. The Hub will help the larger rare disease community navigate critical intersections across the FDA that affect patients with rare diseases, such as medical devices, diagnostic tests, and combination products.
- The Hub will enhance inter-center collaboration to address common scientific, clinical, and policy issues related to rare disease product development, including relevant cross-disciplinary approaches related to product review, and promote consistency across offices and centers.
- The Hub will advance regulatory science with dedicated workstreams for considering novel endpoints, biomarker development and assays, innovative trial design, real-world evidence, and statistical methods.

The FDA’s ALS Science Strategy is disease-specific and builds on current science and research to support drug development. To inform the development of this scientific strategy, FDA subject matter experts have assessed areas of unmet medical need and the current state of clinical studies to identify challenges in the context of drug development, as well as exploring opportunities for purposeful FDA scientific leadership.

To date, FDA partnerships have provided the MIDD tools that help synthesize current knowledge on disease progression and sources of variability, incorporate sophisticated trial design methods, and inform researchers regarding adequate trial sizes and durations through clinical trial simulation (CTS) and sample and effect size estimates based on user-friendly graphical user interfaces via the R Shiny web app [78]. These clinical trial simulators and innovative trial design tools provide a means to plan and explore scenarios for neurological trials. These tools have the potential to help optimize and streamline trial design and stimulate communications between sponsors and regulators about how best to meet the needs of patients with neurological disorders.

Under the CDER and C-Path collaboration, researchers have added a module to the first regulatory-endorsed clinical trial simulation tool for Alzheimer’s disease^1^ to inform the risk of dropouts due to COVID-19. This module estimates the effect of a dropout based on whether it happened early or late in the trial and includes a washout effect for trial participants unable to visit a clinic to receive study treatment. It also simulates the extra variability in measurements that may arise from virtual visits [79].

The two reported tools assist with planning for a platform trial with up to five treatment arms compared to a single control, saving time and resources compared to conducting five separate trials with five separate control arms. The trial’s design allows for an interim analysis for early stopping due to futility [80]. The first tool is a module added to a clinical trial simulation model^2^, which simulates a platform trial for Parkinson’s disease while allowing users to adjust the effect size of each arm separately (Figure 1). The second tool is a disease-agnostic graphical user interface that informs sample size requirements and statistical criteria for trial success and early stopping using the MAMS (multi-arm multi-stage) R package [81].

As many treatments for Parkinson’s disease only affect the symptoms of the disease and not its underlying progression, C-Path also developed a module for clinical trial simulation tool 2 that uses two methods to compare a disease-modifying effect versus a symptom-only effect in a randomized delayed-start trial [82,83].

Traditional clinical trials often use a pre-defined 1:1 or 2:1 allocation ratio, which may result in more participants being allocated to a control treatment than necessary. To inform optimization of the number of trial participants on treatment relative to the control, an MIDD tool was developed that implements response-adaptive randomization, which optimizes the number of trial participants on a treatment while maintaining power and sample size based on prior patient responses to the treatment [84].

Furthermore, there are ethical concerns associated with leaving trial participants with a progressive disease on control medications. To address these concerns, researchers have developed another module for a clinical trial simulation model [85] for Duchenne muscular dystrophy; it allows for control group participants to switch from the control to the active treatment if their progression passes a threshold of endpoint change from the baseline, which can be specified by the user using an inverse probability-weighting approach [86].

These new tools will help facilitate drug development and provide regulators and sponsors with a platform to discuss regulatory submissions, explore various “what if” scenarios, and serve as an educational resource for reviewers. Such MIDD solutions may also help with patient recruitment, as potential trial participants may be more willing to enroll in trials where they can switch to the experimental treatment. Furthermore, the broad adoption of these tools has the potential to reduce the number of in-house tools that regulators need to review. Ultimately, they have the potential to remove bottlenecks in the drug development process for the abovementioned disease areas, thereby making safe and effective therapies available for patients in need.

## 5. Shifting the Focus

The FDA is currently focused on creating new clinical trial protocols, identifying biomarkers, and modeling studies in which the patients are few, dropout occurs, and the effect is marginal. The purpose of supporting these studies is to encourage developers to bring more drugs into testing, essentially removing their uncertainty. However, this goal has not been achieved; now, almost halfway through the FDA’s 5-year plan, there is a dire need to bring more rational thinking to resolve this failure that should have been anticipated, as the bioavailability of drugs which must cross one of the blood–organ barriers is typically limited.

Novel monoclonal antibodies have been developed for the treatment of neurodegenerative diseases (NDs) that operate by crossing the blood–brain barrier (BBB) [87]. Nevertheless, it is commonly reported that their bioavailability in the brain is approximately 0.1% of the quantities found in the bloodstream [78]. Similarly, several studies have found that the extent of central nervous system (CNS) exposure to circulating biologics is restricted to 0.1 to 0.4% of the corresponding concentrations in the bloodstream [88,89,90]. However, some estimates for the transportation of immunoglobulins into neural tissue have been much lower [91].

The determination of sample size in clinical trials depends on several key statistical factors, including the expected effect size, the variability in response, and the desired statistical power of the study. When the anticipated treatment effect is minimal, distinguishing this effect from random variations requires a larger sample size. Effect size is a measure of the magnitude of the treatment response compared to the control group; when the expected effect is large, fewer participants are needed because the difference is more easily detectable. However, when the effect size is small, a larger sample is required to establish this difference with statistical significance [92].

Variability in responses, or the extent to which individual outcomes differ, also plays a significant role in sample size determination. A high variability makes it harder to detect true treatment effects, necessitating a larger sample to ensure that the observed differences are not due to chance. Statistical power, usually set at 80% or 90%, represents the likelihood of correctly identifying a true effect, and achieving this power level requires adjusting the sample size based on effect size and variability [93]. In placebo-controlled trials, the challenge is even greater because the treatment must show a clear difference compared to the placebo. Small treatment effects demand a high number of participants to demonstrate efficacy, especially when placebo effects are substantial, as often seen in trials for neurodegenerative diseases.

Study designs, such as parallel or crossover designs, and the selection of endpoints, also influence the sample size needs. Sensitivity analyses conducted during the planning phase can help assess how variations in assumptions about effect size or variability impact the required sample size, ensuring that the trial in question is adequately powered under different scenarios [93]. Additionally, regulatory and budget constraints influence sample size decisions; larger trials are more expensive, and researchers may mitigate costs by selecting more responsive outcomes or using surrogate markers that require fewer participants. These adjustments ensure that clinical trials are appropriately sized to detect meaningful treatment effects, particularly when a minimal response is anticipated.

**Figure 3:**
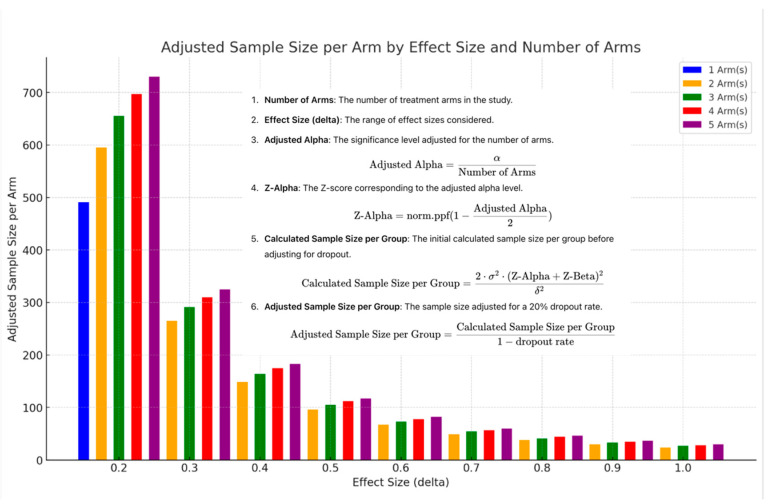
Adjusted sample size per arm by effect size and number of arms. Blue: 1 arm (no control; single-arm study); orange: 2 arms; green: 3 arms; red: 4 arms; and purple: 5 arms [author-created image].

If the bioavailability in the brain (or anywhere across the target barriers) is substantial, the effect will be higher, reducing the study size and increasing the robustness of these trials. Figure 3 depicts how the study size changes with multiple arms and an expanded effect range in a research program that the FDA has supported.

Ideally, rationally and ethically, the clinical trials to test drugs that fall under the ALS Act should only be allowed if there is proof of reasonable bioavailability at the site of action and, thus, an increased chance of the drug proving successful. This validation can be easily carried out through tissue distribution studies in animal models such as rats, as described in this study.

The extent to which a drug returns into systemic circulation can also impact its therapeutic efficacy within the CNS. If a significant portion of the drug is absorbed into the bloodstream, this might reduce its concentration and effectiveness within the CSF and CNS. The drug’s reabsorption into systemic circulation can also lead to systemic side effects, especially if the drug has effects outside the CNS or if high systemic concentrations are reached (Figure 3).

While intrathecal administration delivers drugs directly to the CNS, some drugs will inevitably be absorbed into the systemic circulation through various pathways. Understanding this process is essential for optimizing their therapeutic efficacy, minimizing systemic side effects, and designing appropriate dosing regimens. Pharmacokinetic studies and careful monitoring are necessary to achieve the desired balance between the CNS and systemic drug levels.

**Figure 4:**
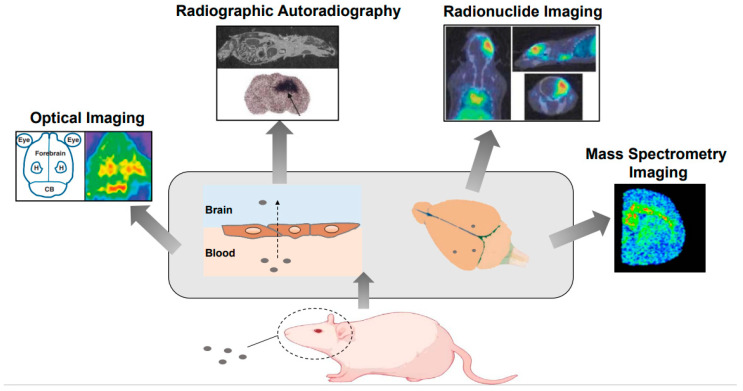
Technologies for imaging.

Determining whether a drug reaches the brain in animal models, such as rats, involves several techniques, including fluorescence imaging, radioactivity-based methods, and tissue sampling. Fluorescence imaging is commonly used when drugs are tagged with fluorescent markers, allowing for non-invasive real-time tracking of the drug’s distribution in the brain through the use of techniques such as two-photon microscopy [94]. Radioactivity-based methods, such as positron emission tomography (PET) or autoradiography, involve labeling the drug with a radioactive isotope, enabling the precise quantification of drug penetration across the blood–brain barrier (BBB) and accumulation in specific brain regions [95]. Tissue sampling, or ex vivo analysis, is another method in which the brain tissue is harvested post administration and drug concentrations are measured using techniques such as high-performance liquid chromatography (HPLC) or mass spectrometry. This approach provides detailed insights into a drug’s distribution within different brain regions but is more invasive than fluorescence- and radioactivity-based methods [96]. These techniques, often combined, allow researchers to comprehensively evaluate the pharmacokinetics and biodistribution of drugs intended to target the brain in pre-clinical studies (Figure 4).

These methods can provide a detailed map of drug localization with high spatial resolution. Developers should be required to submit a comparative analysis with and without transcytosis agents to confirm a change in pharmacokinetic studies. The FDA can create protocols that define how “substantial” bioavailability is designed. However, what is “substantial” shall remain controversial. If the bioavailability is less than 10%, then it is logical to assume that the drug may fail in large-scale clinical use, where the placebo begins to have a significant impact.

Three-dimensional in vitro models have emerged as powerful tools for predicting the absorption of drugs, particularly as they can mimic the complexity of human tissues more accurately than traditional 2D cell cultures. These models, such as organoids and tissue-engineered constructs, can simulate the architecture and microenvironment of various tissues, including the gastrointestinal tract, which is crucial for studying drug absorption and bioavailability. Specifically, 3D blood–brain barrier (BBB) models have been developed to assess the permeability of drugs across the BBB, a critical factor in determining whether a drug can enter the brain. These models typically incorporate human endothelial cells, astrocytes, and pericytes to replicate the in vivo environment of the BBB, allowing researchers to study drug transport, penetration, and efflux mechanisms under physiologically relevant conditions [97].

Studies have shown that these 3D BBB models can effectively predict the entry of drugs into the brain. For instance, research using 3D co-culture systems has demonstrated the ability to assess the permeability of small molecules, peptides, and antibodies across the BBB, providing insights into their potential efficacy for treating central nervous system disorders [98]. While these models offer significant advantages, including the ability to perform high-throughput screening and reduce reliance on animal models, challenges remain in fully replicating the complex dynamics of the human BBB. However, as these models evolve, they promise to improve the predictive accuracy of drug absorption and brain penetration in the early stages of drug development [99].

In addition to 3D in vitro models, additional methodologies such as in situ and silico models are also utilized for the prediction of drug penetration into the brain. Typically, in situ models include isolated perfused brain preparations, such as the brain perfusion technique in rodents, in which the drug is directly administered into the blood vessels of the brain. The utilization of this technique enables the accurate quantification of drug transportation across the blood–brain barrier (BBB) and yields significant information regarding the kinetics of brain absorption and dispersion. The labor-intensive nature of in situ models makes them less appropriate for high-throughput screening compared to in vitro models, despite their great applicability to in vivo circumstances.

In contrast, in silico models utilize computational simulations and machine learning methods to forecast the permeability of drugs across the blood–brain barrier (BBB). These models employ the physicochemical characteristics of the medication, including its lipophilicity, molecule size, and hydrogen bonding potential, in conjunction with data from previous in vitro and in vivo experiments, in order to predict blood–brain barrier (BBB) penetration [89]. Increasingly sophisticated in silico methods, such quantitative structure–activity relationship (QSAR) models, provide more precise predictions and are considered valuable in the early phases of drug discovery for screening extensive libraries of compounds [90].

Moreover, cell-based assays using human-derived brain endothelial cells, often combined with advanced techniques such as microfluidics, can also simulate the dynamic environment of the BBB. These platforms allow for the real-time monitoring of drug transport and interaction with the BBB, offering insights into the mechanisms underlying drug permeability [100]. Integrating these diverse models, including in vitro, in situ, and in silico approaches, provides a comprehensive framework for evaluating the potential of drugs to penetrate the brain, facilitating the development of effective therapies for central nervous system disorders.

## 6. Conclusions

While the FDA is making extensive efforts to help find treatments for NDs, the focus has mainly been placed on study design, not on the formulation of drugs. Given the long history of failures of these drugs, it is now well established that significant bioavailability is essential to demonstrate any efficacy, which can be initially tested in animal models during development. It has also been proposed that the only practical approach to enhancing the bioavailability of a drug in the brain is to adopt the transcytosis approach, preferably involving irreversible entry into the brain to achieve a sufficiently effective concentration. These technologies have matured but are yet to be adopted where they are most needed. In the meantime, the FDA should restrict the approval of INDs, as it has for thousands of such studies, exposing millions of patients to the fate of failed studies that are tantamount to human abuse.

The need for ND treatment is dire, and the US Congress and the FDA are committed to encouraging the discovery of new drugs. However, the approach must be rational and scientific. Fortunately, explicit solutions are available, although strong FDA commitments will be required to bring these to millions of patients.

## Figures and Tables

**Table 1:** Studies reported on clinicaltrials.gov to be in phases I–IV to treat NDs (August 2024).

Phases	Alzheimer’s Disease	Parkinson’s Disease	Lewy Body Dementia	Multiple Sclerosis	Huntington’s Disease	Amyotrophic Lateral Sclerosis
PHASE3	162,784	416	-	351	20	13,094
PHASE2	73,860	1150	5	200,690	69	212,902
PHASE4	33,203	1029	8050	1528	-	598
PHASE1	23,128	3389	116	3132	686	2634
PHASE2|PHASE3	21,136	163	113	-	-	6630
PHASE1|PHASE2	9683	416	30	391	50	1507
EARLY_PHASE1	2189	524	70	60	114	206
Grand total of patients	325,983	7212	8384	206,152	939	237,571
Studies	1583	139	11	111	25	401

## Appendix A

**Table A1:** List of prominent drugs that have failed. The projected penetration rates are less than 0.1%, with most less than 0.01%.

Aducanumab [101]	Monoclonal antibody targeting amyloid-beta plaques. Two phase III trials (ENGAGE and EMERGE) were halted in 2019 after futility analyses.
Alzhemed [102]	A small molecule designed to inhibit amyloid aggregation. Phase III trials failed to show significant cognitive benefits in patients with Alzheimer’s.
Atabecestat [103]	A BACE1 inhibitor aimed at reducing amyloid-beta production. Phase II trials were terminated early due to liver toxicity concerns and lack of efficacy.
Atomoxetine [104]	A norepinephrine reuptake inhibitor typically used for ADHD, tested for cognitive benefits in Alzheimer’s disease. Phase II trials failed to show significant benefits.
Azeliragon [105]	An antagonist of the receptor for advanced glycation end products (RAGE). Phase III trials failed to demonstrate efficacy in slowing cognitive decline in patients with Alzheimer’s.
Bapineuzumab [106]	A monoclonal antibody targeting amyloid-beta. Phase III trials (Study 301 and 302) failed to meet the primary endpoints.
Blarcamesine [107]	A sigma-1 receptor agonist and muscarinic modulator. Phase II/III trials showed mixed results, with no clear cognitive benefits over the placebo.
Bryostatin-1 [108]	A protein kinase C modulator. Phase II trials showed mixed results, with no clear cognitive benefits over the placebo.
Crenezumab [109]	A monoclonal antibody targeting amyloid-beta. Phase III trials (CREAD 1 and CREAD 2) failed to demonstrate significant cognitive benefits.
Eliprodil [110]	An NMDA receptor antagonist intended to reduce excitotoxicity. A phase III trial failed to demonstrate efficacy in improving cognitive function in patients with Alzheimer’s.
Encenicline [111]	A selective alpha-7 nicotinic acetylcholine receptor agonist. Phase III trials (Cognition 1 and Cognition 2) were terminated due to severe gastrointestinal side effects and lack of efficacy.
EVP-0962 [112]	A gamma-secretase inhibitor tested for cognitive improvement in Alzheimer’s disease. Phase II trials failed to show significant cognitive benefits.
Gantenerumab [113]	A monoclonal antibody targeting amyloid-beta. Phase III trials were discontinued due to a lack of efficacy in slowing cognitive decline.
Ginkgo biloba [114]	An extract from the *Ginkgo biloba* tree, tested for cognitive benefits in Alzheimer’s disease. Phase III trials failed to demonstrate significant cognitive benefits.
Idalopirdine [115]	A 5-HT6 receptor antagonist aimed at improving cognitive function. Phase III trials (STARSHINE and STARBRIGHT) failed to demonstrate significant cognitive benefits.
Intepirdine [116]	A 5-HT6 receptor antagonist intended to improve cognition and function. Phase III MINDSET trial failed to demonstrate efficacy.
Ladostigil [117]	A combined cholinesterase and MAO-B inhibitor. Phase II trials showed no significant cognitive benefits in patients with Alzheimer’s.
Lanabecestat [118]	A BACE1 inhibitor aimed at reducing amyloid-beta production. Phase III trials (AMARANTH and DAYBREAK) were terminated due to a lack of efficacy.
Latrepirdine [119]	Originally developed as an antihistamine, Dimebon was repurposed for Alzheimer’s treatment. Phase III trials, including the CONCERT trial, failed to show efficacy.
Lecanemab [120]	An amyloid-beta protofibril antibody. Phase II trials initially showed some promise, but further analysis and trials showed inconsistent results and a lack of robust clinical benefits.
LMTM [121]	A tau aggregation inhibitor. Phase III trials failed to meet their primary cognitive endpoints, though a subgroup analysis suggested potential benefits in non-users of symptomatic therapies.
Levetiracetam [122]	An anti-epileptic drug tested for potential neuroprotective effects in Alzheimer’s disease. Phase II trials did not show significant cognitive benefits.
Liraglutide [123]	A GLP-1 receptor agonist typically used for diabetes, tested for potential neuroprotective effects in Alzheimer’s disease. Phase II trials failed to show significant cognitive benefits.
Ly2886721 [124]	A BACE1 inhibitor intended to reduce amyloid-beta production. Phase I/II trials were terminated early due to liver toxicity concerns and lack of efficacy.
LY450139 [125]	A gamma-secretase inhibitor aimed at reducing amyloid-beta production. Phase II/III trials did not show significant cognitive benefits and were associated with adverse effects.
Masitinib [126]	A tyrosine kinase inhibitor targeting neuroinflammation. Phase II/III trials showed some promise, but the data were inconsistent, and the primary endpoints were not met.
Mefenamic acid [127]	A non-steroidal anti-inflammatory drug (NSAID) tested for its potential to reduce neuroinflammation in Alzheimer’s disease. Phase II trials showed no significant cognitive benefits.
Methylene Blue [128]	Methylene Blue was repurposed as an anti-tau aggregation agent. A phase II trial initially showed promise, but subsequent trials failed to replicate these findings.
Nilvadipine [129]	A calcium channel blocker tested for potential neuroprotective effects. Phase III trials (NILVAD study) failed to show significant cognitive benefits.
Oligomannate [130]	A marine-derived oligosaccharide aimed at reducing neuroinflammation. Phase III trials in China showed initial promise, but further global trials failed to replicate these results.
PF-04494700 [131]	A PPAR-gamma agonist aimed at reducing amyloid-beta production and neuroinflammation. Phase II trials failed to demonstrate cognitive benefits.
Pioglitazone [132]	A PPAR-gamma agonist typically used for diabetes, tested for potential neuroprotective effects. Phase II/III trials (TOMMORROW trial) failed to show significant cognitive benefits.
Ponezumab [133]	A monoclonal antibody targeting amyloid-beta. Phase II trials did not show significant cognitive benefits, leading to the termination of the program.
Posiphen [134]	A small molecule aimed at reducing amyloid precursor protein (APP) levels. Phase II trials did not show significant improvements in cognitive function.
Resveratrol [135]	A naturally occurring polyphenol thought to have neuroprotective properties. Phase II trials did not demonstrate significant cognitive benefits in patients with Alzheimer’s.
Rilapladib [136]	A lipoprotein-associated phospholipase A2 inhibitor that showed promise in reducing cognitive decline in Alzheimer’s disease.
Riluzole [137]	An existing drug for amyotrophic lateral sclerosis (ALS) repurposed for Alzheimer’s. Phase II/III trials did not show significant cognitive benefits in patients with Alzheimer’s.
Saracatinib [138]	An SRC kinase inhibitor repurposed from oncology to Alzheimer’s disease. Phase II trials did not show significant cognitive improvements.
Scyllo-Inositol [139]	An amyloid-beta aggregation inhibitor. Phase II trials failed to demonstrate significant cognitive benefits in patients with Alzheimer’s.
Semagacestat [140]	Gamma-secretase inhibitor aimed at reducing amyloid-beta production. Phase III trials halted in 2010 due to worsening cognition and an increased risk of skin cancer.
Semorinemab [141]	A monoclonal antibody targeting the tau protein. Phase II trials (TAURIEL) did not meet the primary endpoints related to cognitive decline.
Simvastatin [142]	A statin tested for potential neuroprotective effects in Alzheimer’s disease. Phase II trials failed to show significant cognitive benefits.
Solanezumab [11]	Monoclonal antibody targeting soluble amyloid-beta. Three phase III trials (EXPEDITION 1, 2, and 3) failed to show significant clinical benefits.
Edonerpic Maleate [143]	A neuroprotective agent with multiple mechanisms of action. Phase II trials did not show significant benefits in cognitive function.
Tarenflurbil [144]	Anti-inflammatory drug and gamma-secretase modulator aimed at reducing amyloid-beta production. A phase III trial in 2008 failed.
Tideglusib [145]	A GSK-3 inhibitor aimed at reducing tau pathology. Phase II trials did not show significant cognitive benefits in patients with Alzheimer’s.
Tramiprosate [102]	A small molecule designed to prevent amyloid aggregation. Phase III trials failed to show significant cognitive benefits.
Troriluzole [146]	A prodrug of troriluzole aimed at modulating the glutamate levels. Phase II/III trials did not meet the primary endpoints of reducing cognitive decline in patients with Alzheimer’s.
CoQ10 [147]	An antioxidant aimed at reducing oxidative stress in Alzheimer’s disease. Phase II trials did not show significant benefits in cognitive outcomes.
Verubecestat [148]	A BACE1 inhibitor aimed at reducing amyloid-beta production. Two phase III trials (EPOCH and APECS) were terminated in 2017 and 2018.
VTP-43742 [149]	An RORγt inhibitor aimed at reducing neuroinflammation. Phase II trials were terminated due to liver toxicity concerns and a lack of efficacy.
PBT2 [150]	A metal protein-attenuating compound designed to reduce amyloid-beta toxicity. Phase III trials failed to demonstrate efficacy in both Alzheimer’s and Huntington’s disease.
Olatersen [151]	An antisense oligonucleotide targeting SOD1 mutations. Phase I trials showed limited safety, but no significant clinical efficacy was observed in later phases.
Omigapil [152]	A neuroprotective agent that showed positive results in slowing disease progression in patients with ALS in phase III trials.
Tirasemtiv [153]	A fast skeletal muscle troponin activator that showed positive effects on muscle strength in patients with ALS.
Tominersen [154]	Antisense oligonucleotide designed to lower the production of mutant huntingtin protein. The phase III GENERATION HD1 trial was halted in 2021 due to a lack of efficacy and concerns about adverse events.
Nelotanserin [155]	A selective 5-HT2A receptor inverse agonist. Phase II trials for the treatment of visual hallucinations in Lewy Body Dementia failed to meet the primary endpoint.
Amiselimod [156]	A selective sphingosine 1-phosphate receptor modulator. Phase III trials failed to show significant efficacy in reducing disease progression in MS.
Natalizumab [157]	A monoclonal antibody against integrin α4, primarily used in relapsing forms of MS. Phase III trials in primary progressive MS failed to show significant benefits.
Ocrelizumab [158]	A monoclonal antibody targeting CD20 on B cells. While successful in relapsing forms of MS, a phase III trial in primary progressive MS (ORATORIO) failed to meet its primary endpoint.
CEP-1347 [159]	An inhibitor of the JNK signaling pathway aimed at neuroprotection. A phase II/III trial (PRECEPT) was terminated early due to futility.
Glatiramer acetate [160]	An immunomodulatory drug originally used for multiple sclerosis, tested for neuroprotective effects in Parkinson’s disease. Phase II trials showed no significant benefits in motor symptoms.
MitoQ [161]	MitoQ is a mitochondria-targeted antioxidant. A phase II trial aimed to assess its efficacy in slowing the progression of Parkinson’s disease.
Nilotinib [162]	A tyrosine kinase inhibitor originally used in chronic myeloid leukemia, repurposed for Parkinson’s disease. Early trials showed some promise, but subsequent trials failed to demonstrate clear benefits.
Ubiquinone [163]	A mitochondrial cofactor thought to have neuroprotective effects. A phase III trial (QE3) found no evidence of efficacy in slowing Parkinson’s disease progression.
